# Endoscopic ligation (“Loop-And-Let-Go”) is effective treatment for large colonic lipomas: a prospective validation study

**DOI:** 10.1186/1471-230X-14-122

**Published:** 2014-07-08

**Authors:** Hrvoje Ivekovic, Nadan Rustemovic, Tomislav Brkic, Rajko Ostojic, Klaus Monkemuller

**Affiliations:** 1 Department of Gastroenterology and Hepatology, University Hospital Centre Zagreb, Zagreb, Croatia; 2 Department of Internal Medicine, Gastroenterology and Infectology, Marienhospital, Bottrop, Germany

## Abstract

**Background:**

Colonic lipomas (CL) are rare benign adipose tumours usually found incidentally during colonoscopy. Endoscopic resection of symptomatic large CL remains controversial, since significant rates of perforation have been reported. In recent years, a novel technique for removal of large CL has been described, consisting of looping and ligating the lipoma with a nylon snare. The aim of our study was to evaluate the safety and efficacy of the “loop and let go” technique for large colon lipomas in a large case series.

**Methods:**

Consecutive patients referred to our institution for colonoscopy were eligible for the study. The diagnosis of CL was confirmed endoscopically by “pillow” and “naked fat” signs. Following diagnosis, lipomas were looped and ligated by endoloop. Follow-up colonoscopies were scheduled at 1- and 3-months interval.

**Results:**

A total of 11 patients with large CL were enrolled in study. The indications for the colonoscopy included altered bowel habits (7 patients, 64%), screening for colorectal neoplasm (3 pts, 27%) and lower gastrointestinal bleeding (1 pts, 9%). The median lesion size was 3 cm (range 2,5-6 cm). Lesions were located at the hepatic flexure in 4 patients (36%), cecum and ascending colon (4 pts, 36%), rectosigmoid (2 pts, 18%) and transverse colon (1 pts, 9%). There were no immediate and late complications. On follow-up (median follow-up time 11,9 months, range 8–24), there was one small residual lipoma (<1 cm).

**Conclusion:**

The results of this study confirm that “loop-and-let-go” technique is safe and efficacious treatment of large colonic lipomas.

## Background

Colonic lipomas are uncommon, benign adipose tumours of the colon, with a reported prevalence of 0,3% [1]. Nonetheless, they are the most common non-epithelial tumours of the colon and among frequency second to adenomatous polyps [2]. The age at diagnosis ranges within 50–69 years, with no differences in sex prevalence [1-3]. Endoscopically CL are easily recognised as a well-delineated, soft, round or ovoid, yellowish sessile or pedunculated mass, originating from the submucosa in 90% of cases [1-4].

Most of CL are asymptomatic, and are usually found incidentally during colonoscopy, surgery or autopsy [1-5]. However, those larger than 2 cm can cause change in bowel habits or abdominal pain - due to intermittent intussusceptions from prolapse or obstruction [1,3-5]. In addition, lesions larger than 4 cm may lead to perforation, or gastrointestinal bleeding due to ulceration of the lesion [1,2,4,5]. Extremely uncommon, transformation to liposarcoma has been documented [6,7].

In these circumstances, surgical or endoscopic resection is warranted [8,9]. However, endoscopic resection of large CL is controversial, because of priore reports of a high rate of perforation [10]. It is postulated that lipomatous tissue contains low water content and therefore conducts electrosurgical current less efficiently [11]. Thus, increasing the power to assist the endoscopic resection may lead to increased heat production and damage to the adjacent bowel wall with subsequent perforation [12].

In recent years, a novel technique for removal of large CL has been described, consisting of looping and ligating the lipoma with the detachable snare [13-15]. By using this technique, the need for electrocautery is avoided, thus eliminating the risk of perforation or bleeding. We have been using this technique for several years. However, as this approach warrants further validation, and the literature on the topic is still limited, we undertook a study to verify this technique in our group of patients with large CL. The aim of our study was to evaluate the safety and efficacy of the “loop and let go” technique for large colon lipomas in a large case series.

## Methods

### Patients and study design

This was a prospective, single-centre open cohort study conducted at the University Hospital Centre, Zagreb, Croatia during April 2009 to March 2011. The Institutional Review Board at the University Hospital Centre Zagreb approved the study and the informed consent was obtained from all patients. All patients referred to our institution for colonoscopy were eligible for the study. Exclusion criteria included patients younger than 18 years of age and patients in whom the informed consent was not provided. Data on age, sex, indication and colonoscopy findings were collected. If a large CL was found during colonoscopy, location and size were recorded, and attempt was made to loop and ligate the lipoma. Descriptive statistics were used to analyse the results.

### Endoscopic technique

**Figure 1:**
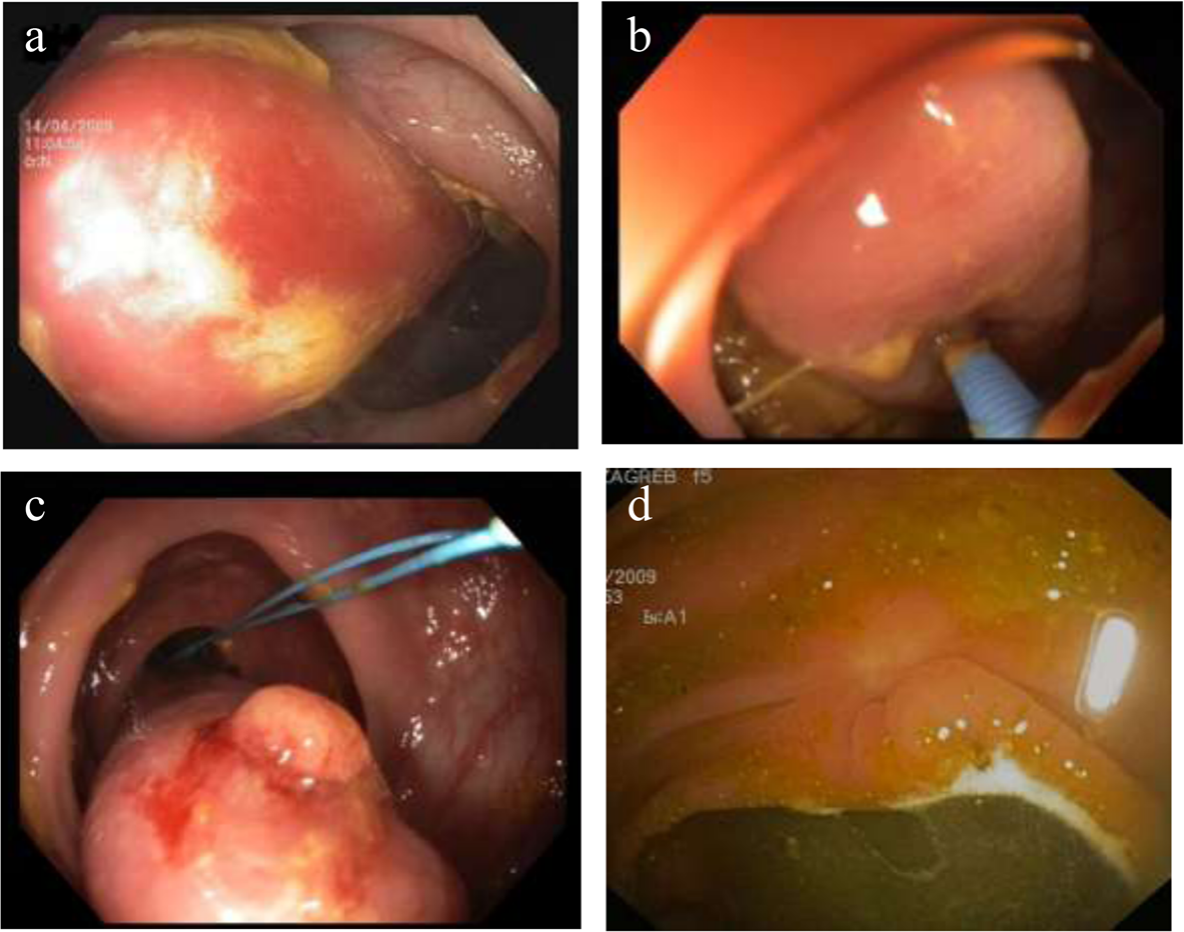
Endoscopic ligation of large colonic lipoma: a) large colonic lipoma situated at the hepatic flexure; b) demonstration of “pillow sign”; c) endoloop deployed at the stalk of the lipoma (“naked fat sign” seen upfront); d) scar seen at the follow-up colonoscopy at 12 weeks.

On the day prior to colonoscopy, patients were instructed to ingest a liquid diet and prescribed 2 bottles of magnesium sulfate. Unsedated procedures were performed, with a standard colonoscope (GIF - H180, Olympus, Tokyo, Japan). The diagnosis of CL was made endoscopically by “pillow” sign (indentation of the lesion on probing with a closed biopsy forceps) and “naked fat” sign (extrusion of the fat after repeated biopsies done at the same location). Only large lipomas discovered in symptomatic patients, regardless of anatomic location or the size of its base, were considered for endoscopic ligation using the “loop and let go” technique (Olympus Endoloop, Olympus Tokyo, Japan) as described by Kaltenbach et al. [15] (Figure 1). The following symptoms were considered to be positively or possibly associated with lipomas: lower gastrointestinal bleeding, intussusception, and lower abdominal pain. In addition, if the polyp had signs of erosions or recent bleeding it was considered for ligation. A large CL was defined as a lesion being >2 cm in size, measured by open biopsy forceps. In brief, the nylon snare of the endoloop was inserted into the lumen and manoeuvred as to capture the head of the lipoma and carefully advanced to the stalk. The plastic sheath of the endoloop was then slowly advanced for controlled closure, and then the snare was completely tightened around the stalk. If needed, prior to the placement of the endoloop, the patient was repositioned as to suspend the lipoma and to obtain the best view of the base or the stalk. All procedures were performed by experienced endoscopists. The assistant nurse handling the loop had previous experience using this device and had deployed more than 50 before the beginning of our study. Follow-up colonoscopies were scheduled at 1- and 6-months intervals, and depending on the indication on the outset of the colonoscopic procedure.

## Results

During study period (April 2009 – March 2011) a total of 6929 colonoscopies were performed in 5563 patients. A total of 11 patients with the large CL were identified (0,02% of all patients) and enrolled in study. Baseline patient characteristics are shown in Table 1. There were 6 males (55%), and the median age was 68 years (range 58–70). The indications for the colonoscopy included altered bowel habits (7 patients, 64%), colorectal carcinoma screening (3 patients, 27%) and lower gastrointestinal bleeding (1 patient, 9%). The median lesion size was 3 cm (range 2–6 cm). Lesions were located at hepatic flexure in 4 patients (36%), cecum and ascending colon (4 patients, 36%), rectosigmoid (2 patients, 18%) and transverse colon (1 patient, 9%). We did not encounter any difficulties during placement of any endoloop. All patients had an uneventful post-procedure course. On follow-up (median follow-up time 11,9 months, range 8–24), there was one small residual lipoma (<1 cm).

**Table 1:** Baseline patient characteristics (N = 11)

**Characteristic**	**n/N (%)**
**Male**	**6/11 (55)**
**Age (median, range)**	**68 (58–70)**
Indication:	
Altered bowel habits	7/11 (64)
Colorectal carcinoma screening	3/11 (27)
Lower gastrointestinal bleeding	1/11 (9)
Location:	
Cecum and ascending colon	4 (36)
Hepatic flexure	4 (36)
Transverse colon	1 (9)
Rectosigmoid colon	2 (18)
Lipoma size (median, range)	3 (2–6)

## Discussion

In this prospective cohort study we demonstrated that the “loop and let go” technique using the endoloop is an effective endoscopic therapy for large CL. Although there are other studies documenting this technique, our study stands out for being prospective and encompassing a relatively large number of patients. Indeed, we are not aware of any larger study using this technique. Thus, our data add to the existing literature on the utility of endoloop to treat submucosal tumors.

Since its initial development by Hachisu, for prevention of delayed bleeding following snare polypectomy of large polyps with thick stalks, detachable snares have become an important part of the endoscopic armamentarium in a variety of therapeutic procedures [16]. Ligation with detachable snare has been successfully deployed in the arresting and control of bleeding of oesophageal [17,18], gastric varices [19], GIST [20], as well as in the management of the large penduculated submucosal tumors [21-23] and ileal lipoma [24]. Despite these publications the utilisation of endoloop for treating submucosal lesions has been mainly reflected by single case reports or small case series [25].

Although the term “loop and let go” is a misnomer, since it is the virtue of ligation and not the looping that produces an asymptomatic, slow mechanical transection of the lesion, the term has gained far more acceptance in the literature, than the proper form of “ligate and let go”.

In general, endoloop ligation of gastrointestinal lesions without resection is conceptually simple and has the potential to eliminate the risk of perforation or bleeding inherent to the electrocautery. However, only those lesions capable of resulting in problems and complications should be targeted, whereas lipomas smaller than 2 cm and asymptomatic warrant no treatment [15].

There are some drawbacks of the ligation technique in regard to the management of large symptomatic colonic lipomas. In order to effectively ligate the lipoma, the stalk needs to be clearly seen, preferably at 5–7 o’clock position. If not, reposition of the patient is required, which may be cumbersome in some situations. Furthermore, the endoscopic ligation should not be attempted in the treatment of broad based or sessile colonic lipomas [12]. In these circumstances, endoscopic or surgical resection may be appropriate. Finally, a potential disadvantage of this technique is that the entire lesion is not recovered for histopathologic evaluation.

There are promising reports of a successful endoscopic resection of large CL [12,26]. However, we believe that this technique has several potential advantages over exisitng methods of endoscopic removal as the “loop and let go” technique avoids the risks associated with electrocautery, potential less hospital stay and theoretical advantage of less perforation. Specifically, when dealing with fatty tissue and lipomas, any endosocpist has certainly endured the difficult situation of having to apply massive amounts of currents to finally resect this lesions.

Our study has potential limitations. First, it is a relative small number of patients from a single center. However, this study represents one of the largest series using the technique of “loop-and-let-go” reported so far. Second, the threshold limit that we used was 2 cm, since, the data in the literature suggest that polyps larger than this isze are more likely to cause problems [11]. Furthermore, we only included symptomatic patients. Finally, our centre is a tertiary university medical centre and the results may not be reproduced in smaller endoscopy units. However, we believe that any skilled endoscopist should be able to master this technique.

## Conclusion

We conclude that the “loop-and-let-go” technique is safe and effective in the treatment of large CLs. We believe that results of our study add to the growing body of knowledge in regard to endoscopic ligation of large submucosal lesions.

## Competing interests

The authors declare that they have no competing interests.

## Authors’ contributions

HI was involved in study concept and design; acquisition, analysis, and interpretation of data; and drafting and revising the manuscript. NR and TB were involved in study supervision and revising the manuscrip. RO and KM were involved in revision of the manuscript. All authors contributed to and approved the final manuscript by providing constructive suggestions.

## Pre-publication history

The pre-publication history for this paper can be accessed here:

http://www.biomedcentral.com/1471-230X/14/122/prepub

## References

[B1] Rogy MA Mirza D Berlakovich G Winkelbauer F Rauhs R Submucous large-bowel lipomas-presentation and management. An 18-year study Eur J Surg 1991 157 51 55 1675882

[B2] Vecchio R Ferrara M Mosca F Ignoto A Latteri F Lipomas of the large bowel Eur J Surg 1996 162 915 919 8956963

[B3] Amer NM Johnston D Gutmann J Image of the month. Intussusception caused by lipoma of the colon Arch Surg 2006 141 833 16924092 10.1001/archsurg.141.8.833

[B4] Krishnan SJ Shehab TM Strasius SR Giant colonic lipoma presenting as intermittent obstruction Clin Gastroenterol Hepatol 2006 4 xxv 16527684 10.1016/S1542-3565(05)00863-3

[B5] Kitamura K Kitagawa S Mori M Haraguchi Y Endoscopic correction of intussusception and removal of a colonic lipoma Gastrointest Endosc 1990 36 509 511 2227328 10.1016/s0016-5107(90)71128-5

[B6] Gutsu E Ghidirim G Gagauz I Mishin I Iakovleva I Liposarcoma of the colon: a case report and review of literature J Gastrointest Surg 2006 10 652 656 16773759 10.1016/j.gassur.2005.09.014

[B7] Chen KT Liposarcoma of the colon: a case report Int J Surg Pathol 2004 12 281 285 15306943 10.1177/106689690401200312

[B8] Kim CY Bandres D Tio TL Benjamin SB Al-Kawas FH Endoscopic removal of large colonic lipomas Gastrointest Endosc 2002 55 929 931 12024158 10.1067/mge.2002.124098

[B9] Tamura S Yokoyama Y Morita T Tadokoro T Higashidani Y Onishi S ‘Giant’ colon lipoma: what kind of findings are necessary for the indication of endoscopic resection? Am J Gastroenterol 2001 9 1944 1946 11419863 10.1111/j.1572-0241.2001.03909.x

[B10] Pfeil SA Weaver MG Abdul-Karim FW Yang P Colonic lipomas: outcome of endoscopic removal Gastrointest Endosc 1990 36 435 438 2227312 10.1016/s0016-5107(90)71110-8

[B11] Bahadursingh AM Robbins PL Longo WE Giant submucosal sigmoid colon lipoma Am J Surg 2003 186 81 82 12842756 10.1016/s0002-9610(03)00111-9

[B12] Geraci G Pisello F Arnone E Sciuto A Modica G Sciumè C Endoscopic Resection of a Large Colonic Lipoma: Case Report and Review of Literature Case Rep Gastroenterol 2010 4 6 11 21103220 10.1159/000260053 PMC2988890

[B13] Raju GS Gomez G Endoloop ligation of a large colonic lipoma: a novel technique Gastrointestinal Endosc 2005 62 8 988 990 10.1016/j.gie.2005.08.018 16301055

[B14] Friedland S Kahng LS Torosis J Soetkino RM Ligate and let go Gastrointest Endosc 2003 58 3 473 474 14528239 10.1067/s0016-5107(03)00039-7

[B15] Kaltenbach T Milkes D Friedland S Soetikno R Safe endoscopic treatment of large colonic lipomas using endoscopic looping technique Dig Liver Dis 2008 40 12 958 961 18434264 10.1016/j.dld.2008.03.010

[B16] Hachisu T A new detachable snare for hemostasis in the removal of large polyps or other elevated lesions Surg Endosc 1991 5 70 74 1948617 10.1007/BF00316840

[B17] Shim C Cho J Park Y Kim YS Kim YT Hong SJ Moon JH Cho YD Kim JO Kim YS Lee JS Lee MS Mini-detachable snare ligation for the treatment of esophageal varices Gastrointest Endosc 1999 50 673 676 10536326 10.1016/s0016-5107(99)80019-4

[B18] Hepworth C Burnham W Swain C Development and application of endoloops for the treatment of bleeding esophageal varices Gastrointest Endosc 1999 50 677 684 10536327 10.1016/s0016-5107(99)80020-0

[B19] Cipolletta L Bianco M Rotondano G Piscopo R Prisco A Garofano ML Emergency endoscopic ligation of actively bleeding gastric varices with a detachable snare Gastrointest Endosc 1998 47 400 403 9609435 10.1016/s0016-5107(98)70227-5

[B20] Brkic T Kalauz M Ivekovic H Endoscopic hemostasis using endoloop for bleeding gastric stromal tumor Clin Gastroenterol Hepatol 2009 7 9 e53 e54 19361578 10.1016/j.cgh.2009.04.003

[B21] Lee SH Park JK Park do H Chung IK Kihm HS Park HS Kim SJ Cho HD Endoloop ligation of large pedunculated submucosal tumors (with videos) Gastrointestinal Endosc 2008 67 3 556 560 10.1016/j.gie.2007.10.049 18294522

[B22] Hashiba K D'Assunção MA Armellini S Hassegawa RT Cappellanes CA Moribe D Esophageal leiomyoma from the muscularis propria treated by ligate and let go technique [abstract] Gastrointest Endosc 2004 59 AB4

[B23] Binmoeller KF Loop-and-let-go for gastric GIST. Daveproject Avialable at: http://daveproject.org/loop-and-let-go-for-gastric-gists/2009-06-01/, visited: January 7,2013

[B24] Veloso R Pinho R Rodrigues A Pais T Fernandes C Carvalho J Fraga J Enteroscopic “loop-and-let-go” ligation of an ileal lipoma by balloon-assisted enteroscopy Endoscopy 2012 44 E176 22622730 10.1055/s-0031-1291752

[B25] Murray MA Kwan V Williams SJ Bourke MJ Detachable nylon loop assisted removal of large clinically significant colonic lipomas Gastrointest Endosc 2005 61 6 756 759 15855988 10.1016/s0016-5107(04)02650-1

[B26] Jovanovic I Pavlovic A Popovic D Pavlov M Endoscopically removed giant submucosal lipoma Vojnosanit Pregl 2007 64 6 417 420 17687948 10.2298/vsp0706417j

